# A Discourse on the Causes, Effects, and Preventives of Intemperance, from Ardent Spirits

**Published:** 1828-04

**Authors:** Daniel Drake


### WESTERN JOURNAL

OF THE

### MEDICAL & PHYSICAL SCIENCES.

APRIL, 1828.

### ESSAYS AND CASES.

Art. I.—*A discourse on* Intemperance; *delivered by appointment, at a public meeting of the Agricultural society of Hamilton County, Ohio, March, 1st, 1828, and subsequently pronounced, by request, before a popular audience.* By Daniel Drake, M. D.

"The pestilence that walketh in darkness."

### Sect. I.—Of the Nature of Ardent Spirits.

Scarcely any one is ignorant of the fact, that when vegetable substances containing sugar, are broken down and mixed with water, and the compound is left to stand in a moderate degree of heat, an intestine motion takes place, and the ingredients undergo a variety of changes. This is vinous fermentation. If the mixture, at a certain stage of this process, is subjected to distillation, a transparent fluid, lighter than water, and of a peculiar, hot and penetrating taste, is obtained. This liquor is Ardent or Distilled spirits; and consists, essentially, of water, and another fluid, imparting the properties, which are expressed by the word ardent or burning. In popular works, and in treatises on the Arts, this ingredient is called spirit of wine, because it was originally obtained by distilling that liquor; but the chemists denominate it Alcohol, a word adopted into the languages of Europe, from the Arabians, who at a certain time were addicted to the study and practice of Chemistry. Alcohol, strictly speaking, does not exist in nature. It is formed out of three of the elementary ingredients of plants, carbon, oxygen, and hydrogen, united with each other in definite proportions, by the process of fermentation. Thus it is present and makes a constituent part of wine, beer, cider, and, indeed, of almost every fermented beverage. But in all of them, it exists in a much smaller proportion than in whiskey, brandy, rum, and other distilled spirits; and is moreover disguised by the presence of undecomposed sugar, several different acids, the bitter principle of the hop and other ingredients which are absent from distilled spirit, because they are not so volatile as to rise in distillation. Ardent spirits, then, are alcohol diluted with water; and the various kinds of fermented drink are alcohol, still more diluted, and at the same time abounding in other substances, which give them a complicated and nutricious character.

It is well known that some one or more of these liquors, are generally and copiously drunk by almost every nation. In one country we find wine the national drink, in another beer, in a third cider,—in the Western Country it is the spirit distilled chiefly from rye and Indian corn. Other drinks are in use, but the quantities consumed are comparatively so small, that this may fairly be considered our national beverage, and to its use and abuse I shall limit my inquiries.

### Sect. II.—Of the Mode of Operation and effects of Ardent Spirits.

The various reasons given for the use of ardent spirits imply, that they are a hot and stimulating drink; and all experience testifies, that in large draughts they act as an irritant, and, finally, prostrate the strength to which they gave a momentary activity. These facts clearly prove, that they are an agent of great power. Their influence reaches, indeed, to almost every part of the body When taken in moderate quantities by those who are not accustomed to their action, theyexcite, in the stomach, a glow, which is sometimes pleasurable, at others painful: the heart is made to beat with more frequency and force, so that the blood circulates with greater liveliness through all the organs: the brain feels the genial impulse, and, immediately, begins to perform its functions in a higher tone: motion, sensation, feeling and thought, become more active, and for a time the whole man, both in soul and body, is animated beyond his usual state; but if no other portion be swallowed, he soon falls back to that condition. If, however, while the effect of the first dose is still upon him, he should take a second or more, a temporary fever is excited. His pulse beats boisterously, his feelings and actions become intemperate, his courage is transformed into rashness, and his trains of thought move onward in gay and giddy disorder. No more being swallowed, this condition, in a short time, gives place to one of languor and inefficiency; but if additional draughts be taken, the symptoms of high excitement yield to those of debility, perverted sensation, and idiotic or brutal delirium. At last, a total inability to stand or even sit, a feeble and irregular pulse, vomiting, stupor and deep sleep, terminate the paroxysm; and, passing off, leave the individual in a state of exhaustion, from which he does not fully recover under two or three days. Such are the effects of copious draughts of ardent spirits taken at short intervals. If a large portion be swallowed at once, instant death may be the consequence. Thus, Mr. Brodie, a distinguished English surgeon, found that a table spoonful of alcohol thrown into the stomach of a rabit, would destroy life in a few minutes. But we are not dependent on experiments of this sort, for proofs, that ardent spirits are a deadly poison; for the fatal accidents which they occasion, in society, are unfortunately too numerous. A striking fact of this kind, was once communicated to me, by the late Dr. Fredrick Ridgely of Kentucky, who belonged to the medical staff of our revolutionary army. A soldier, when so far restored from a fever, as to walk about the esplanade of the fort, drank, at a single draught, half a pint of whiskey, and instantly fell down dead! Other melancholy accidents, in our own times, might be referred to with advantage; and I will mention one, which shows not only the fatal power of ardent spirits, but what is of more importance at this moment, the way in which their habitual use, by some, may occasion the death of others. In Lexington, a few weeks since, a poor widow with three children, happening to reside near a cotton bagging factory, observed the operatives to drink freely, and was induced to procure a barrel of whiskey, and retail it to them, as a means of contributing to the support of her small children. But a sad consequence ensued. Within a short time, when she herself, was gone to market to purchase food, they drew, and in imitation of her customers, drank freely of the poison. When she returned they were all postrate, and in a few hours one of them was a corpse.

It is however, unnecessary, to multiply facts, to establish the proposition, that ardent spirits are a great quickener and disturber of the animal system; a warm and irritating poison; in moderate doses imparting an unnatural excitement; in excessive draughts suddenly extinguishing life. In these respects obeying the same law with many deleterious vegetable substances—such as stramonium, hemlock, the prussic acid and opium—which we label as poisons, and place beyond the reach of the imprudent and the ignorant.

Sect. III.—Of the Necessity for using Ardent Spirits.

Having briefly stated the nature and effects of ardent spirits, we come, in the third place, to inquire whether their habitual use is necessary to the preservation of health, and to the due performance of the offices of human life. No one, unperverted by intemperance, could be hardy enough to answer this question in the affirmative; for such a reply would involve a down right absurdity. Ardent spirits are *not* one of the natural and necessary supporters of life. He who created man in infinite wisdom, could not have made it a condition of his healthy existence, that he should resort, daily, to an agent of artificial origin, and of such power that it might suspend his activity, or even put an end to his existence in a single hour. Bread and meat, warmth, air and water, are the appointed sustainers of animal life. It is scarcely possible to use them to excess, and without them we languish and die.

God has implanted in us a desire for them, because they are necessary to our existence and well being. Hunger and thirst are natural appetites; but ardent spirits can satisfy neither. True, they will for a short time suspend the cravings of hunger, and finally destroy the appetite for food; but they produce these effects by exciting in the stomach an irritation, which is in fact a real disease. Thus instead of satisfying our desire for sustenance, ardent spirits *annihilate* it:—an admirable piece of service to a poor fellow, shipwrecked upon a barren rock in the midst of the ocean; but a very different affair, to an inhabitant of the fat and fruitful banks of the Ohio, where a good appetite can detract from the happiness of nobody. Ardent spirits, indeed, impart no nourishment; and he who might be reduced to short allowance, would live longer without than with their pretended aid, though he might live less merrily.

Nor will they quench thirst. The natural appetite, calls for water only. Water makes more than nine tenths of the blood, and all the other fluids of the body; and this proportion must be maintained or we suffer decay of health. Hence the disposition to drink is perhaps the deepest rooted of all our desires; and, when not gratified, it becomes, at last, the most tormenting. But the desire is not for ALCOHOL. It is for water; and nothing *but* water can satisfy it. True it is, that other fluids are sometimes substituted; but water makes the basis of the whole, and they are efficacious, in quenching thirst, and supplying the wants of the system, in proportion to the quantity in which that fluid enters into their composition. Thus brandy, gin, and whiskey; the different kinds of wine, beer, porter, lemonade, punch, milk, whey, chocolate, tea and coffee, contain a proportion of water varying from one half to ninety nine hundredths, and it is in virtue of this *water*, and *not* of the ingredients which it holds in solution, that they are able to quench thirst. Indeed, with the single exception of the native vegetable acids, which some of them contain, the water that enters into their composition, is more efficacious in allaying thirst, if given in its purity, than when thus adulterated with foreign matters. Ardent spirits then remove thirst, and supply the waste of the system, by means of their *water* instead of their *alcohol;* and, thence, in reference to our natural desires and wants, the latter is unnecessary and indeed inapplicable.

But although not required nor qualified, to satisfy either hunger or thirst, and, therefore, not called for under ordinary circumstances, *extraordinary* occasions are said to require the use of ardent spirits. For the sake of argument, let us suppose such cases to exist. If they do, is the *habitual* use of ardent spirits, the way to give them *occasional* efficacy? This is, indeed, the most certain method of rendering them inert and useless, in the emergencies that are said to demand their aid. Becoming accustomed to their action, the system is rendered insensible to their animating influence, when it is most needed. If our paths are beset with perils, which we suppose cannot be passed through without the aid of an artificial stimulant, let us reserve it, till we reach the time and place of difficulty. The soldier who might exhaust his magazines upon a distant enemy, would make but a sorry defence, when the foe should approach within striking distance; and still he would not act more absurdly, than the man who attempts to fortify himself against *occasional* danger, by *habitual* stimulation.

But is it a *fact*, that ardent spirits *can* sustain us under exposures and emotions, to which we should yield without their agency? I do not hesitate to say, that, in general, their aid is altogether imaginary, while in many cases, their treacherous influence, sides with the enemy—which a delusive hope whispered to us, they would vanquish. Let us now briefly inquire into some of the cases which are said to call for the use of ardent spirits.

I. Exposure to cold. Habitual drinking, so far from protecting us against cold, really, makes us much more liable to injury from it. The drunkard is, indeed, peculiarly tender, as it respects cold. But when the temperate are subjected to it, should *they* resort to strong drink? If the exposure is brief, such a resort may be permitted; but is then, in general, unnecessary. If it be protracted, I see no reason to believe that its pernicious effects can be warded off by the stimulus of ardent spirits. That agent, it is true, can increase for a time the generation of heat in the body; but the law of the animal system is, that if actions are raised above their natural degree by artificial means, they afterwards fall below it—so that he who can brave the cold, in the hour of intemperate indulgence, sinks under it in the hour of weakness that follows. He is alternately less and more vulnerable than the man, who, mounting to no delusive elevation, suffers no dangerous depression; but moves forward on a uniform and safer level.

2. Exposure to intense heat. This is always attended with danger; but ardent spirits cannot diminish the hazard. Too much heat is retained in the system, because the hot atmosphere conducts off too little; and why should we increase its production by a stimulating drink? The true preventives are water, vegetable food [abundant in warm climates,] and rest in shaded situations, during the heat of the day. With these, ardent spirits are superfluous—without them pernicious.

3. Exposure to moisture. Wet clothes, a damp atmosphere, fogs, moist ground, and water, conduct off the heat of our systems with great rapidity, even if the weather and water are not actually cold. Under this kind of exposure, the exciting influence of ardent spirits is thought, by almost every body, to be indispensable to the preservation of health. The remarks just made are applicable, with but little variation, to this case. If the exposure be slight and casual, a single draught may do good; but if prolonged, or constantly recurring, the system must be supported by other agents, less transient, and less irritating in their effects. Of these, active exercise, a diet of animal food made savoury with salt and other condiments, strong coffee or tea, and the application of great warmth to the feet at night, are the means on which we may rely with most confidence.

4. Fatigue is said to render ardent spirits necessary. It may be admitted that intemperate persons cannot make great exertions, or endure fatigue, without drinking; but with the utmost aid of their boasted stimulant, they are unable to perform so much as mere water drinkers. The exceptions to this remark are only apparent. Many powerful men are addicted to the excessive use of ardent spirits, and are thought, by superficial observers, to owe their bodily strength to that which their firm constitutions enable them to resist. They may be said to continue strong, in spite of the enervating effects of dram drinking. But the stoutest will, at last, yield to that, which promptly subdues the weak; and neither acts wisely, to resort to an agent, which is superfluous to the strong, and useless to the feeble. Both, however, may derive assistance, in a single effort, from ardent spirits, provided their systems are not habituated to the action of that liquor; but when long continued labours are to be performed, both are injured by the unnatural stimulation. The repetition of the dose, never fails to irritate the inner surface of the stomach, and that irritation as infallably produces weakness.

Thus it is quite obvious, that those who are exposed to cold, moisture, or heat, or are compelled to make great or long continued exertions, cannot derive any substantial aid from the use of ardent spirits. They should not, therefore, resort to them, because they may thus contract a *habit* of drinking; and all who do this, whatever may have been their native resolution, are liable in the end to fall its victims. In support of these various conclusions, every observing man must have met with many individual examples. As triumphantly establishing the first, I may refer to the case of those who survey our public lands. No persons in the community endure such fatigue, or are so much exposed—in summer to burning heat, when traversing extensive prairies; in winter to intense cold; and, in spring and autumn, to cold and moisture combined,—for they often wade through snow and half frozen marshes for whole days, and lodge for months on the damp ground;—and yet, as I have been assured by Mr. Arthur Henrie, a surveyor of observation and much experience, they in general enjoy excellent health, tho' destitute of the pretended aid of ardent spirits. The number of persons who have, within the last 25 years, been employed in surveying the national domain, between the gulf of Mexico and the Lakes, has amounted to several thousand; and a more conclusive experiment, therefore, could not well be made.

Hence it appears that those who resort to ardent spirits, as a means of support under exposure and great fatigue, commit a most fatal error. The labouring men of the country, who are often required to make great exertions, and are exposed to cold heat and moisture—market people—butchers—waggoners and draymen—boat men, subjected to fogs and the dampness of night—post riders and stage drivers—country physicians, who, in thinly settled places, are compelled to make long professional journeys at night, under every inclemency of the weather—fishermen—stone-quarriers and well diggers—labourers on our canals, lodged almost in the open air—salt-manufacturers—forge, furnace and foundry men—finally, hard labourers, operatives, and mechanics of every sort, should be solemnly and affectionately warned, that when, under exposure or in great exertion, they rely on the aid of ardent spirits, they "lean upon a broken staff." Of their hard and honest savings, they give a part—some times a *large* part—for that which gradually undermines the vigour of body they would preseve, and on which all their success in life perhaps depends. Many of them are the victims of this false theory, whose suggestions so coincide with their appetites, that they can be saved, only by being instructed. The task is one of difficulty and discouragement, but we should not be dismayed. It is the duty of physicians to disclose the truth—of philanthropists to enforce its adoption.

5. Certain perplexed or painful states of mind are thought to require, or at least to be palliated by ardent spirits. Thus, it is supposed to lend salutary aid to the exhausted student, whose ardour still glows, when the flame of his midnight lamp has expired;—to the merchant or capitalist, distracted by the cares and casualties of an extensive, complicated and precarious business;—to those whom folly or misfortune has suddenly thrown from a higher estate;—to physicians, when compelled to devote the natural hours of sleep to inquiry, deep thought, and painful anticipation, over the bed side of the sick and dying;—finally to the mourner, who rejecting all companionship, but that of his own sorrows, holds communion with the dead throughout the livelong night; or wandering abroad, amidst all the gaieties of society, or all the grandeur and beauty of nature, still feels himself in solitude, and perceives around him only a dreary waste. Could ardent spirits sustain and cheer us under these trials, the most scrupulous would scarcely prohibit their use; but alas! reason declares them inadequate, and universal experience confirms the decision. For the regrets and inquietude of human life, they are the most fatal resort that human ingenuity has ever suggested. Their use converts perplexity into confusion, and adds fuel to the consuming influences of grief; it solves no problem, wards off no impending misfortune, and rolls permanently away, not a single cloud of sorrow;—but, adding irritation of body to agony of soul, it dooms to swifter destruction, the unfortunate wretch who trusts to its power.

6. Lastly, several chronic diseases are supposed by the people and some physicians, to require the daily use of ardent spirits. These are chiefly the long and frightful train of distempers, known under the names of dyspepsia, hypochondraism, hysteria, palpitation of the heart, and weakness of the nerves. They prevail, especially from the 20th to the 40th year, and affecting multitudes of both sexes, are fairly entitled to the "bad eminence" of detracting more largely from the happiness of refined society, than any other class of the diseases which civilization has inflicted on us. Infusing poison into every cup of enjoyment, and rearing spectres in all our paths;—transforming courage into cowardice,—and that period of our lives, which on the plan of nature should be most gay and enterprising, into a state of debility and gloom,—it is not wonderful that we are tempted to resort to the potent stimulus of ardent spirits. But of the danger of such a resource, no medical man ought to entertain a doubt—though there may be a few cases in which it is salutary. Weakness, it is true, is a prominent circumstance in these affections; but weakness is, in general, the *consequence* of disease, and can only be removed by its cure. Ardent spirits are a medicine, but in such maladies, they are not the remedy in which we should confide. The transient relief which they afford, is followed by permanent aggravation, and those who rely upon it sacrafice the future to the present—unconsciously, acting with the folly of the man who wastes by anticipation, his most precious resources.

### Sect. IV. Of the Causes of the Abuse of Ardent Spirits.

It is often asked, whether we have a natural taste for ardent spirits? I answer, that we have not; nor have we a natural taste for any thing, which, like them, is purely artificial. We have a natural taste for water, and the productions of the earth; but not for Alcohol, which is the offspring of their decomposition, in the laboratory of the distiller. Alcohol, the intoxicatign ingredient of all our beverages, is itself, indeed, one of the most poisonous productions of art; and it is only by great dilution with water, and the artifice of combining it with more savoury articles, that we can be induced to swallow it.

But if we have no natural taste for alcohol, why do so many persons take it to excess? I reply, that although we have no natural love for that liquor, we have an innate propensity to the use of stimulants, and the excitement which they produce is acceptable to us. Within moderate limits it is harmless, and under proper circumstances even useful. It constitutes the poetic fervour of our physical sytsems. All our organs will act, and do their duty in a regular business style, without being thus excited; but when moderately stimulated, they work more cheerfully, and we are conscious of the difference. It is this consciousness that prompts us to the habitual use of stimulants, both to the body and the soul; for, in reference to the excitement of which I have spoken, the soul and body obey the same law. Thus the newsmonger, the inveterate novel reader, the glutton, and the wine bibbler, conform to a common principle of our nature. In different countries the modes in which we stimulate the body are different. The Arabs resort to coffee, the Chinese to tea, the Turks to opium, the French to wine, the English to ale, the Icelanders to rancid fish oil, and we Backwoodsmen to *Whiskey.* For this variety there are causes, some of which are obvious and may be mentioned. Thus coffee is a native of Arabia; tea of China; wine is forbidden in Turkey; the vine flourishes in France; barley and hops attain to great perfection in England; and rye and Indian corn furnish us sturdy republicans with whiskey at so cheap a rate, that the poorest man in the community can get drunk, as often as his wealthiest neighbour:—so that our equality, by no means depends on the precarious nature of our boasted political institutions.

Among individuals, there is a still greater variety in the manner and means of stimulation, than among nations. Thus, to draw our examples from home, some regale themselves on tobacco—solid, powdered, or gaseous; others flourish in the midst of political broils; and a few sustain themselves by the stimulus of interminable litigation: the excitement of gaming in its seductive varieties of cards, horse racing and billiards, is delightful to many; others prefer the stimulants of a table loaded with savoury viands; some could enjoy themselves quite as much, on public executions and horrible accidents, if they would, fortunately, but occur often enough; others are content to read tales of mystery and marvel, provided they are not diluted with the realities of useful knowledge; the thrilling transports of musick captivate a few; tea and coffee exhilirate many more; here and there, we find an individual, unhappily, enamoured with the stupifying influence of opium; finally, a considerable number relish only madeira, champaign, or constantia wine; a more numerous body guzzle porter and strong beer; but the sheet anchor of the multitude is *Whiskey.*

Under this great apparent diversity of taste and practice, it is not difficult to perceive, that the end in view is nearly the same—the stimulation of our organs, feelings, and faculties into a higher state of excitement. It is for this we have a native taste—an inherent desire—a constitutional longing, which exists from birth, and expires, only with the vital spark itself. To this principle, we may ascribe excessive drinking, and almost every other intemperate indulgence. If *it* were destroyed, drunkenness would disappear from the land. This, however, would be to change, not the condition merely, but the constitution of human nature—an object neither to be accomplished nor desired. We cannot extinguish the propensity which leads to intemperance; but may limit and regulate its indulgence; and to this our efforts should be directed. Let us proceed, then, to inquire into the causes which give inordinate energy to this principle, that, by avoiding them, we may preserve ourselves from the disasters of its unrestricted influence. In this country, they are chiefly the following:

1. Habitual drinking. This is, manifestly, a great cause of drunkenness. Custom reconciles us to things the most offensive; then renders them agreeable; and ends by exalting them into objects of uncontrollable desire. Thus, by repetition we are made to relish equally the savor and the effects of ardent spirits; and, at last become drunkards, from taste as well as constitution. I am aware that there are many who believe that habitual drinking does not on the whole lead to excessive indulgence, but the facts are against them. On this question the practice of the society of Friends is conclusive, and deserves universal imitation. The rules of that respectable community proscribe the use of ardent spirits except as a medicine, and wherever the proscription has been enforced, drunkenness has nearly disappeared. In Cincinnati, for a quarter of a century, so far as I can recollect, I have had but one patient of that society, whose disease was referable to intemperance; and at present, when the number of Friends is very considerable, there is, in the whole city, but *one* addicted to intoxication. It is in vain to look within the pale of any other congregation for a similar state of sobriety; they all denounce drunkenness, but tolerating the daily use of ardent spirits, the denunciation is too often of no avail.

Among the varieties of daily drinking two or three deserve to be held up to public reprobation. One of these may be called family drinking, and, in its least injurious form, consists in handing round among children, what are provincially called *juleps*, *toddies* and *slings*; or other domestic mixtures containing alcohol, and rendered agreeable with sugar and aromatics. When confined to the evening this practice is objectionable, but extended to the earlier parts of the day it is pernicious. I know there are discreet and temperate parents who are its advocates, and argue, that if a child is accustomed to such drinks from its infancy it will long for them less when it grows up, than if it had been debarred from their use. But the same parents would not allow their sons to practise any other vice, lest they should contract a fondness for it. They do not distinguish, between permitting children to taste of ardent spirits, unmixed with "palatable sweets," and the daily use of them with those pleasant additions. It must be granted, that families reared under this absurd usage often remain temperate; but when there is any strong native propensity to sensual gratification, intemperance can scarcely fail to occur.

The fashion of offering ardent spirits to our ordinary visitors, is fraught with still greater mischief, and cannot be too soon abolished. Unfortunately it is so general that few men in society have the consideration or courage to violate it; and, in obedience to the same fatal rule of social intercourse, still fewer have the courage to drink only when they are dry.

The habits of landlords and bar keepers deserve a passing remark. Many intemperate men take to these occupations as furnishing the means of support and indulgence, when all other resources are dried up; but granting all this, it is undeniable, that an extraordinary proportion of these people become drunkards, from the custom of drinking, hourly, with those who frequent their establishments. Thus he who is an accessory to the *felo de se* of so many others, at length destroys his own life.

The intemperance which, in the United States, has prevailed in my own profession, may be considered here. It has been ascribed to the fatigue and watching which physicians, occasionally, are obliged to sustain, and a portion of it is, undoubtedly, referable to those causes; but it is chiefly owing to their custom of drinking, daily, at the houses of sick persons Physicians are, or should be, welcome visitors; and in a country where ardent spirits are a daily offering of hospitality, if they drink at all, they are liable, at length, to drink too much. No physician should allow himself to use ardent spirits, until he has passed his fortieth year. Customs of any kind, commenced after that period, are seldom carried to excess; and neither the profession of medicine, nor society at large, would afford so many drunkards, if this salutary rule were observed. I am happy to be warranted in saying, that in our cities the number of intemperate physicians is fewer than it formerly was; tho' in the villages and the country, they are still so numerous, as to inflict on the profession a disgrace which is truly mortifying. I know of no class of citizens whose examples are more authoritative than those of medical men—especially in what relates to modes of living:—hence a weighty responsibility rests upon them in this particular; and every considerate man, in society, must unite in the hope, that they will hereafter practice greater self denial.

But of the various kinds of daily drinking, the most fatal is morning drams. At that hour, the system is more sensible to the action of stimulants, than at any other; and the custom of adding bitters to the ardent spirits, greatly increases the danger of future intemperance; for the more offensive the taste of any substance, the more bewitching is our attachment when once established. Indeed no man ever made daily morning visits to the splendid bar-room of a city hotel, or the homelier shelves of a country tavern, without being, or becoming, a drunkard. The rising sun, which lights the unsteady footsteps of such a man to the polluted fountain, shall not set early enough to hide his shame. If he be unmarried, let every mother exclude him from her habitation, as she values the peace and character of her daughters;—if married, then should his wife and children prepare, in sadness, for poverty and deep mortification;—if he be in debt, his creditors should beware;—if he make new engagements, expect them not to be fulfilled;—place a stigma on his forehead, and let all society withdraw their confidence and communion, that the virtuous may not be led astray by his example, nor the imprudent brought to ruin in his fall.

2. The use of ardent spirits in assemblies of men convened for business or amusement. Public meetings contribute greatly to intemperance. It signifies, little, for what purpose they are held, or of whom composed, if drinking be permitted, it associates itself with the occasion, and ingrafts the sensual upon the social enjoyment. Some will intoxicate themselves because, a good opportunity offers; others in honor of the meeting; and others through a laudable ambition not to be excelled. This one takes an extra glass, because he has met a friend; that one because he expects to meet an enemy. The old indulge themselves, for the purpose of rising to the mean heat of the party; and the young, out of that respect for age, which characterizes the youth of America. Thus, if all do not exceed the limits of sobriety, it is not the fault of the occasion, which so happily provides an excuse for every one.

The number of meetings, in the United States, at which drinking is permitted, is far greater, than those who have not made an estimate, would suppose. The public dinners in celebration of memorable events, and in honor of great men, ought, in reference to the morals of the nation, be made fewer, however deeply the interests of liberty might suffer by the reduction. Nearly connected with these meetings in object and effect, but on the whole more pernicious, are the barbecues of the West and South, at which the people, to a certain extent, exchange habits of industry and temperance, for politicks, conversation, and merry making; and lose the government of their appetites, in learning to "govern themselves."

Annual horse racing is another occasion for drinking and dissipation. The reason assigned for this public amusement, is directly connected with the agricultural interest. It is said, to encourage the growth of fine horses. But this, with most of those who promote racing, is only a pretext. The real end is to create a strong excitement; which, in general, is attained, whatever may be the fate of the pretended object. But supposing that object to be real and attainable; no moralist or patriot can refuse to condemn racing, unless he adopt the maxim, subversive of all morality, that the end will justify the means. The olympic games required discipline of body, and mind; and, to a certain extent, gave that energy to both, for which the ancient Greeks were distinguished. But these modern games call out the mass of our young men, and array them against each other over the bottle, as their coursers are matched upon the turf. We thus sacrifice the morals of our sons, to improve the speed of our horses!

Intemperance is promoted by several kinds of stated, popular meetings, which are indispensable to the constitution of our free governments. Our annual elections, semi-annual militia trainings, and in Virginia and Kentucky, the monthly courts of justice, are all of this kind. In Ohio the militia musters are most promotive of dissipation; but in Kentucky, from a difference in the laws, all three, are, perhaps, equally injurious. Every philanthropist should open his eyes to the dissipation, which is practised, by many of those who attend on these occasions. They cannot be abolished, but the insobriety which they generate, might be much abated by the frowns and exhortations of the temperate; and it is truly deplorable that these should be so often withheld. Great, however, as the drinking on election days really is, it amounts to nothing, in comparison with the aggregated intemperance, which, in some parts of the Western country, grows out of what is denominated the electioneering campaign. Even in common years, the candidates take the field several months before the election;—when a true politico-civil war ensues, and rages, with increasing violence, till the balloting takes place. The intellectual gladiators are found in array against each other, on every public occasion; and when opportunities do not occur, as frequently as they desire, special meetings are appointed. At these the people assemble, some to criticise, others to be instructed, others to encourage the combatants, and others to drink of that, which is so bountifully poured out to all. Throughout the whole period of feverish excitement, the thirst of the multitude waxes greater and greater; and hundreds drink to excess, who under a different mode of conducting the canvass, would pursue their honest occupations, in peace and temperance. All this is perceived and deplored by many of the candidates; but the custom is uncontrollable. Being a species of public institution, no individual can resist its influence; and sustained by the taste and passions of the people, they only, can effect its overthrow.

3. Gambling. Vices are gregarious, that is, in the language of agriculture, go in flocks. They obey the universal law of association; and hence gaming, knavery and drunkenness are, sooner or later, found united in the same individual. Without inquiring into the process by which gaming generates intemperance, it is sufficient to know the fact. Were this the only consequence, then, that flows from that detestable custom, a sufficient motive would exist for discouraging young persons from the use of cards and every other species of rivalry, that is sustained by betting. I am sorry to know that this is not done; but that the very reverse is the practice of many, who have the superintendence of young persons. They are taught cards and billiards, as innocent amusements, but told not to play for money; which argues a criminal ignorance of the human character. No passion so speedily fattens on its indulgence as gaming; and the young man who at first sat down to *amuse* himself with an *innocent* game of cards, in the company of elegant associates, and surrounded by the refinements of the drawing room, may die among the benches of a vulgar tavern—a desperate gamester and a drunken outcast. A love of gambling once excited, the dissipation which it may generate is not to be foretold by the sagacity of the wisest, nor averted by the prayers of the most devout; but the wisdom and piety of the land ought to unite in a sentence of condemnation, should their utmost efforts but palliate the mischief.

4. Trades and occupations which, daily, bring numbers of men into close connexion, are causes of intemperance. Hatters, tailors, shoemakers and millwrights, with many others, are in this condition. Among 8 or 10 persons thus assembled, there will, generally, be two or three veterans, who consider drinking an essential duty of human life; and take special care, by precept and example, to urge it upon their youthful comrades. Thus a love for strong drink is often generated, in the apprentice, faster than a love for the duties and rewards of the trade; and he ends his novitiate, a botch in every thing but intemperance.

5. Smoking segars. This custom, which is both sensual and social in the enjoyment it confers, unquestionably promotes drinking, and should be abolished. When young men assemble, whatever may be the object, convivial feelings spring up, and they are apt to become thirsty. This condition is, manifestly, aggravated by the smoking in which they then indulge, for it renders the mouth and throat dry. Moreover, tobacco disturbs the nervous systems of most young persons to such a degree, that the stimulus of ardent spirits is, in some measure, necessary to sustain or restore them. As they consider it disgraceful not to break themselves into the habit of smoking, if they once make the attempt, it must be accomplished at every hazard; and the greater constitutional repugnance, the deeper the drinking. But as tobacco is poisonous and ardent spirits remedial, as one imparts painful and the other pleasurable sensations, a fondness for the latter is often generated, long before the system has submitted to the impress of the former. This, however, is only a part of the injury, which results from smoking, for it contributes to dyspepsia and the frightful train of nervous disorders, which prey upon our young men, and call for the correcting influence of public sentiment. The nation has an interest in the physical constitution of its youth, and should look to their habits, with still greater care, than the husbandman watches over the saplings, which, in future, are to constitute his timber trees. Those who have passed the meridian of life, are seldom injured by smoking; but at twenty, the systems of most *American* young men are too sensible, to bear the action of tobacco with impunity.

6. Matrimonial unhappiness is a cause of intemperance. It is unfortunate, that so many wives mistake scolding for remonstrance; and drive their husbands into dissipation by indulging in the former, when a proper exercise of the latter, would contribute so largely to preserve them from every kind of vice. That many a weak minded husband *has* been scolded into drunkenness, is undeniable; but others—or their friends for them—have ungenerously ascribed their intemperance, to the scolding, which it had in reality provoked. At best, moreover, it is but a contemptible excuse for getting drunk, that a man is married to a *scold*, or in other words to a silly, petulent and garrulous woman. It should rather animate him to increased sobriety, resolution and steadiness; that the peace and character of his family may, as far as possible, remain unimpaired. Husbands, in general, exert more influence over wives, than wives can over them; and it would better comport with their superior pretensions, to labour in the correction of a wayward temper, than to render themselves insensible to its outbreakings, by the stimulus of ardent spirits. An intemperate wife, who might assign her husbands moroseness as a reason for her drinking, would find but few persons of either sex, disposed to admit the validity of her excuse. The same rule should be applied to both.

7. Confectionaries and retail groceries. The multiplication of drinking establishments affords evidence of an increasing taste for ardent spirits; but, it is equally certain, that in turn they present new opportunities, and stronger temptations to excessive indulgence. It is melancholy to observe the resort to these sinks of tippling and dissipation. It begins with the dawn of day, and ends not even with midnight. Among the devotees, we see persons of all ages and of every calling, many of whom are overwhelmed with sottishness, before they are publickly known to have departed from the paths of sobriety. Of all the causes of intemperance none act more insidiously than this, and the irremediable drunkenness which it generates, should be more dreaded than cancer or hydrophobia; for their victims expire in innocence, amid the sympathies of society; while the sot too often outlives his character, fortune and friends, to die in obscurity and disgrace.

8. Distilleries. The increase of small distilleries, in the country, gives an effect similar to the increase of groceries in the towns and villages. When a new distillery is erected in a neighbourhood, an additional establishment for idleness and drinking, is opened, to all, whom curiosity, the desire for company, or the love of drink, can draw from their homes and honest occupations. But this is no more than a part of the mischief. Distilleries are the only places, where whiskey can be procured, in barter, for the grain out of which it is made; and there can be no doubt, that this facility greatly promotes the consumption of that beverage. When a man can obtain a gallon of whiskey, by sending to the distillery a bushel of Indian corn, he is more likely to procure it, than if it could be purchased but with money. In an agricultural country, where grain is abundant and money comparatively scarce, men part with the latter more reluctantly than the former. Hence I cannot but regret, in common with many patriotic country gentlemen, the multiplication of little distilleries; and, especially, the custom of bartering small quantities of whiskey for grain.

9. Lastly, the construction of canals, turnpike roads, and other public works, is a cause of intemperance. Thousands of labourers are employed on these national undertakings, and it is deeply to be regretted, that in most if not all parts of the United States, it is the practice of the contractors, to distribute ardent spirits among them, repeatedly, every day. The pernicious effects of this custom need no kind of illustration; and it should be discontinued, as it is absurd for a nation to project works of public utility, and so conduct their execution, as to corrupt their morals, and thus enfeeble the state. It is the duty of those who superintend these national enterprises, to consider what correctives may be appled to an evil of such increasing magnitude; and some of them, I am happy to know, have perceived the force of this obligation, and feel disposed to adopt every means of prevention which the case may admit.

Other causes of intemperance exist in the Western states: I shall not, however, prosecute the unwelcome task any further; but enter on the more pleasant duty of enumerating those which have either disappeared, or are greatly mitigated.

1. The Indians wars, which prevailed extensively, on both sides of the Ohio, from the peace of 1783, to the treaty of Greenville in 1794, contributed greatly to the intemperance of the early settlers. The standing army, sent to the west, was very considerable, in proportion to the whole population; and, in addition to the *Regulars*, almost every person capable of bearing arms, was, at one time or other, ordered into the field. Many young men, indeed, became a sort of professional soldiers; by repeatedly hiring themselves, as substitutes, for others who chose to fight by proxy, or, could not leave home, when drafted. Throughout the whole of these wars the practice of drinking was only limited by the scarcity of whiskey; and after their termination, the military, being dispersed among the civil, carried into society an extraordinary proportion of intemperate men, among whom were many distinguished for wealth, or talents, or public services.

The excessive drinking which characterized the western settlements in their infancy, did not begin to abate till 12 or 15 years after the return of those who had been engaged in their defence, and was undoubtedly in part perpetuated by their example. A striking feature of the intemperance of that period, was its clamorous and unblushing display. It was no disgrace and scarcely any discredit, to appear reeling and boisterous in the public streets—and drinking at the close of dinner parties, balls and even weddings, was sometimes carried through all the successive stages of gaiety, mirth, and uproar, to a quiet and comfortable state of dead drunkenness! Thus opened the 19th century; but at the end of the first quarter, the aspect of society is entirely changed. The generation, who, in the midst of the greatest privations and perils, so gallantly defended, the infant Territories, has nearly passed away; and the example of their failings is already extinct; while the story of their heroism will be told to animate our children for years to come.

But several other causes of intemperance were in active operation at that time, of which the following were the most efficient.

2. In the peopling of the forest, cabins were to be erected, and the trunks of fallen trees rolled together and burnt. These indispensable objects, required men to assemble in large companies; and few employments are more laborious, than what they were obliged to execute. Ardent spirits, of course, were considered necessary; and while they were liberally drunk by the whole, not a few never failed to take them to excess. In the *central* parts of Tennessee, Kentucky, and Ohio, from the progress of settlement, these public occasions have nearly ceased, and with them, of course, the vulgar intemperance by which they were disgraced; but much of it still exists among the frontier population.

3. In the West, particularly to the south of the Ohio River, the wheat and Indian corn harvests were, once, so conducted, as to occasion great insobriety. Each, was made a frolick, and the latter especially, a drunken frolick; which generally ended in absolute riot. I am happy to be able to say, that, from a salutary change in our agricultural customs, this species of dissipation has nearly disappeared.

4. In the earlier periods of our western settlements, tea, coffee, chocolate and various spices were scarce, and of more difficult attainment than whiskey. Cider and beer were, moreover, almost unknown. Hence men were under the necessity of relying, more than at present, on ardent spirits for the stimulation which, to a certain extent, is necessary to our moral and physical activity; and thus were liable to drink to excess.

5. Formerly, several thousand persons were employed on the barges, and keel and flat boats, which navigated the Ohio and other western streams; and no class of people exhibited so much, or such gross dissipation. The age to which they belonged was not very temperate, and the occupation itself naturally attracted the viscious, who being absolved from every moral restraint speedily became much worse. From the progress of society, such of them as remain have improved in their habits but their number is now, greatly reduced; for the small craft which were their homes, have been nearly superseded by steam boats, and the profligate inhabitants have disappeared with the habitations. Thus the genius of Fulton has contributed largely to dispel the moral darkness of our waters, as the sun dissipates the fogs of morning; he has made us better as well as richer; and exhibited to the world, the power of a mechanical invention, in the suppression of immorality and vice.

6. Lastly, the pioneers of a new country are less under the dominion of the laws, both of God and man, than their descendents who compose a denser state of society. In new settlements men have less regard for the opinions of others, and less respect for themselves, than in advanced states of society. Practical moralists and intelligent and pious Divines, are proportionally fewer, than in older communities like our own.

Thus several moral restraints, were formerly wanting in the West which have been since suppliedand; their absence, undoubtedly, was a negative cause of intemperance.

Other causes may have ceased within the last 25 or 30 years, but I have enumerated the principal. They suggest the question, whether intemperance itself has declined? On this subject I have recently asked the opinions of several intelligent friends, both of Ohio and Kentucky, and the observations of the majority coincide with my own:—That drinking at public meetings of every kind, and composed of all sorts of people, is far less than it formerly was; and that drunkenness, both public and private, of the wealthier classes—agricultural, professional and mercantile—has decidedly abated; though still, in all the towns and cities, there are numerous *gentlemen*, who indulge in habitual and quiet stimulation, till they become sots. But there is reason to fear, that among the labouring classes, the practice of daily drinking has increased rather than diminished; and of this melancholy fact it is not difficult to find an explanation. It is manifestly owing, to the abundance and cheapness of distilled spirits, and to the increase of tippling houses. In former times, the high price of whiskey, opposed a barrier to its use among the poor, which the progress of agriculture and the art of distillation has broken down; and all experience demonstrates, that men will indulge themselves to greater excess, as the means are brought more within their command. Men, at that time, could not afford to drink, incessantly; and they reserved the indulgence for convivial meetings: at present, they associate it with their daily labours, and literally make a business of getting drunk. This is the crying evil of the land; and is not limited to the people of the West, for it prevails extensively among the labouring classes of the East.

[*The remaining sections of the Discourse, on the personal and public effects of Intemperance and on the remedies both preventive and curative, will be given in the next number.*]

